# Development of Inhalable Chitosan-Coated Oxymatrine Liposomes to Alleviate RSV-Infected Mice

**DOI:** 10.3390/ijms232415909

**Published:** 2022-12-14

**Authors:** Jianqing Peng, Qin Wang, Mingyang Guo, Chunyuan Liu, Xuesheng Chen, Ling Tao, Ke Zhang, Xiangchun Shen

**Affiliations:** 1 The High Efficacy Application of Natural Medicinal Resources Engineering Center of Guizhou Province, School of Pharmaceutical Sciences, Guizhou Medical University, Guiyang 550025, China; 2 The Key Laboratory of Optimal Utilization of Natural Medicine Resources, School of Pharmaceutical Sciences, Guizhou Medical University, Guiyang 550025, China; 3 The Key and Characteristic Laboratory of Modern Pathogenicity Biology, School of Basic Medical Sciences, Guizhou Medical University, Guiyang 550025, China

**Keywords:** oxymatrine, RSV, chitosan, inhalation, liposomes

## Abstract

Human respiratory syncytial virus (RSV) infection is the most important cause of acute lower respiratory tract infection in infants, neonates, and young children, even leading to hyperinflation and atelectasis. Oxymatrine (OMT), originating from natural herbs, possessed potential antivirus activity against influenza A virus, Coxsackie B3 virus, and RSV, whereas the absence of an in vivo study indicated the difficulties in overcoming the physiological obstacles. Since RSV basically replicated in lung tissue, in this study, we fabricated and characterized a chitosan (CS)-coated liposome with OMT loaded for the treatment of lethal RSV infection via inhalation. The results uncovered that OMT, as a hydrophilic drug, was liable to diffuse in the mucus layer and penetrate through the gas–blood barrier to enter systemic circulation quickly, which might restrict its inhibitory effect on RSV replication. The CS-coated liposome enhanced the distribution and retention of OMT in lung tissue without restriction from mucus, which contributed to the improved alleviative effect of OMT on lethal RSV-infected mice. Overall, this study provides a novel inhalation therapy for RSV infection, and the CS-coated liposome might be a potential inhalable nanocarrier for hydrophilic drugs to prevent pulmonary infections.

## 1. Introduction

Human respiratory syncytial virus (RSV) is a human-originated orthopneumovirus that circulates in winter and spring worldwide. RSV presents a high tropism to the pulmonary system of preterm infants, neonates, and young children, it being the number-one cause of acute lower respiratory tract infection (ALRTI), i.e., pneumonia or bronchiolitis, and severe conditions such as asthma [1]. RSV infection is also a significant cause of morbidity and mortality in the elderly, immunocompromised individuals, and patients with chronic underlying illnesses [2]. Moreover, the specific mechanism for the pulmonary disease is poorly dissected; direct viral cytotoxic injury companied with the pathogenesis of RSV was thought to be a main cause for necrosis of the epithelial cells of the small airways and systemic extrinsic apoptosis of CD4^+^ and CD8^+^ T cells [3,4]. Consequently, such sloughed cells and mucus plug the bronchioles, and thereafter, hyperinflation and atelectasis occur [5].

Matrine-type alkaloids are a group of monomeric compounds that were isolated from *Sophora flavescens* Ait (*S. flavescens* Ait, “Kushen” in Chinese) by a Japanese group in 1895 [6]. They are well known, with a wide range of pharmacological effects such as antiviral, antioxidant, antibacterial, antitumor, antifibrotic, and insecticidal properties [7]. Oxymatrine (OMT) is the representative alkaloid of *S. flavescens* Ait, as investigated in several reports, including against the influenza A virus (IAV), by inactivating the promoters of *TLR3*, *TLR4*, *TLR7*, *MyD88*, and *TRAF6* of host cells and deregulating IAV infection-induced activations of NF-κB, Akt, ERK1/2, and p38 MAPK pathways [8], and against the Coxsackie B3 virus by ameliorating myocarditis in vivo described by our group [9]. Impressively, Ma et al. presented the anti-RSV infection effect of OMT in vitro, which preliminarily demonstrated the anti-RSV activity with promising selectivity index in comparison with ribavirin [10]; however, there is an absence of an in vivo studies. Effective in vivo delivery of OMT to pulmonary infection lesions might be a promising strategy to alleviate RSV-infected mice.

Pulmonary administration has the priority in the treatments of pulmonary infectious diseases, due to it being free from first-pass effect and direct delivery of a loaded drug to lung lesions [11]. However, challenges remain in the pulmonary delivery of therapeutics to infected regions. OMT must overcome numerous physiologic barriers, including enzymatic degradation, mucociliary clearance, pulmonary surfactant absorption and macrophage phagocytosis to achieve pulmonary accumulation [12,13,14]. In the exploration of inhalable drug delivery system against infectious lung diseases, liposomes with great biocompatibility and wide applicability hold a strong promise in improving the stability and cellular uptake of loaded drugs in the respiratory tree, especially for hydrophilic drugs [15,16]. OMT liposomes have been reported for several decades, and they were mainly applied on liver diseases, including acute liver injury [17], liver fibrosis [18], and hepatocellular carcinoma [19]. In addition, OMT liposomes were applied in the treatment of ulcerative colitis and intervertebral disc degeneration as well [20,21]. In our previous work, OMT liposomal preparations have been developed for the treatment of emphysema via pulmonary administration [22]. Although carboxymethyl chitosan (CMCS) coating slightly enhanced the pulmonary retention of positively charged liposomes, the fast systemic distribution of OMT liposomes, regardless of the CMCS coating, indicated the necessity for further optimization of the prescription.

Enlightened by the constitution of endogenous pulmonary surfactants, we prepared OMT liposomes (Lipo/OMT) by 1,2-dipalmitoyl-sn-glycero-3-phosphocholine (DPPC), hydrogenated soybean phosphatidylcholine (HSPC), and 1,2-dipalmitoyl-sn-glycero-3-phospho-(1′-rac-glycerol) (DPPG) in this study instead of soyabean lecithin, in order to improve its stability in vivo. To further enhance the pulmonary delivery, a surface modification strategy was used to prevent the degradation and immune recognition of liposomes and facilitate the mucus penetration [23]. 1,2-distearoyl-sn-glycero-3-phosphoethanolamine-N-methoxy (polyethylene glycol) (DSPE-PEG) has been widely used to escape from immune surveillance and facilitate mucus penetration via hydration shell, whereas as a double-edged sword, PEGylation hindered the interactions with cells at the same time [24]. Given that chitosan (CS) has been widely used in the construction of nanocarrier for pulmonary delivery as both matrix and shell due to its high biocompatibility and biodegradability [25,26], it is reasonable to circumvent the “PEG dilemma” via coating of CS on the periphery of PEGylated OMT liposomes. Both high-molecular-weight (30 kDa) and low-molecular-weight (800~1000 Da) CS were adopted to prepare the surface-coated liposomes (Lipo/OMT@CS). The optimal type of CS and the weight ratio of CS/liposome was optimized in terms of the characteristics of liposomal preparations. Both Lipo/OMT and Lipo/OMT@CS were used for the treatment of RSV-infected mice via inhalation.

**Figure 1:**
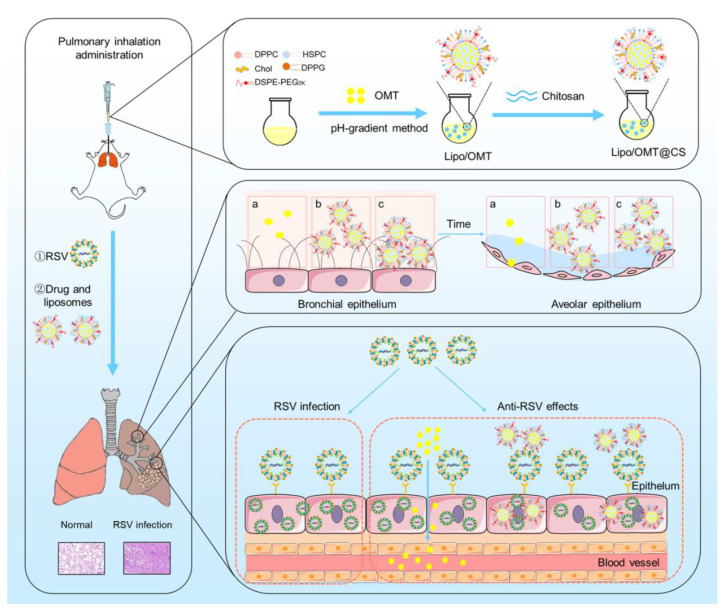
Schematic illustration of the preparation process of CS-coated OMT liposomes, the distribution after inhalation and the inhibitory effect on RSV infection (a: free OMT, b: Lipo/OMT, c: Lipo/OMT@CS).

In this study, we developed inhalable CS-coated OMT liposomes for the treatment of RSV-infected mice. The optimal CS-coated OMT liposomes were evaluated in vitro and applied on the RSV-infected mice model in which the anti-RSV mechanisms of OMT might be attributed to its preventive effects on RSV (Figure 1). This study provides an effective liposomal platform for pulmonary delivery of hydrophilic therapeutics against viral infection.

## 2. Results and Discussion

### 2.1. Inhibitory Effects of OMT on RSV-Infected HEp-2 Cell

**Figure 2:**
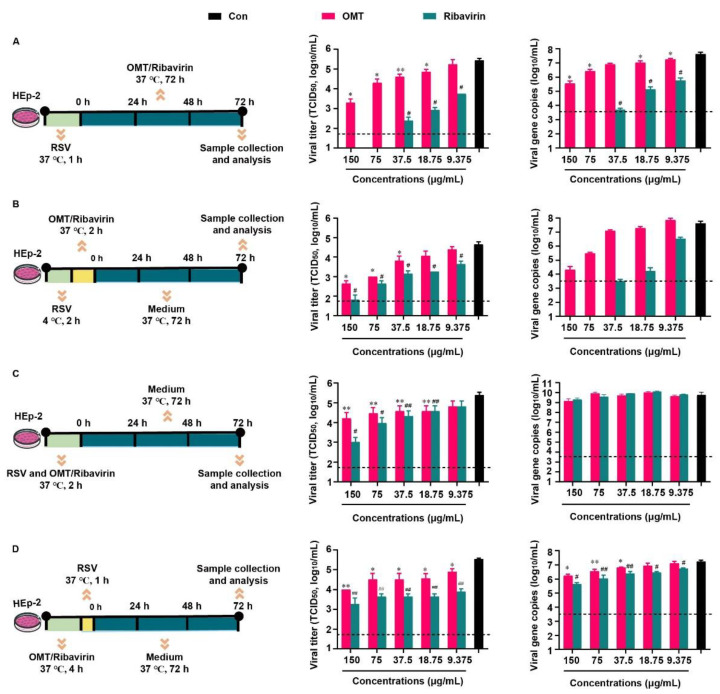
Inhibitory effects of OMT on RSV infection and replication in HEp-2 cell. The experimental scheme, TCID_50_ and qRT-PCR result of (**A**) virus biosynthesis assay, (**B**) virus penetration assay, (**C**) virus attachment assay, and (**D**) drug pre-treatment assay. All the data were expressed as mean + SD (*n* = 3). * and ** represent OMT compared with virus control (black bar) as *p* < 0.05 and *p* < 0.01; # and ## represent ribavirin compared with virus control (black bar) as *p* < 0.05 and *p* < 0.01. The dash line indicates the limit of detection in TCID_50_ assay and qRT-PCR assay, respectively. Con: virus control.

Based on the previous report that related to the inhibitory effect of OMT on RSV in vitro. Above all, the cytotoxicity of OMT and ribavirin on HEp-2 cells were evaluated (Figure S1), and the non-cytotoxic concentrations of OMT and ribavirin were used in the following study. We evaluated the inhibitory effect of OMT and ribavirin on RSV from 4 aspects. They were virus biosynthesis (Figure 2A), virus penetration (Figure 2B), virus attachment (Figure 2C), and drug pre-treatment (Figure 2D). Since RSV was capable to enter cells quickly, it was incubated with HEp-2 cells, at 37 °C, for 1 h when no drug was presented in the medium (Figure 2A,D). In the study of virus penetration, RSV was pre-incubated with HEp-2 cells, at 4 °C, for 2 h to achieve sufficient binding before penetration (Figure 2B). In terms of inhibition on the virus attachment, RSV was mixed with drugs ahead of co-incubation with HEp-2 cells, and thus, a longer incubation time of 2 h at 37 °C was used (Figure 2C). Based on the TCID_50_ assay result, measurements of 150 μg/mL, 75 μg/mL, 37.5 μg/mL, and 18.75 μg/mL OMT were found to significantly decrease viral titers and viral gene copies indicating the intervention effects of OMT on the virus biosynthesis (Figure 2A). Ribavirin showed more excellent anti-RSV effect than OMT as virus biosynthesis inhibitor. According to the TCID_50_ result, 150 μg/mL, 75 μg/mL, and 37.5 μg/mL OMT significantly blocked the penetration of virus into cells (Figure 2B), which was confirmed by the result of viral gene copies. Ribavirin showed more excellent anti-RSV effect than OMT as a virus penetration inhibitor. In the study of the virus attachment, OMT and ribavirin showed weak anti-RSV effect. No statistical differences in both OMT and ribavirin was observed in terms of viral gene copies (Figure 2C). After pre-treatment of OMT, the viral titer and gene copies were slightly reduced, which was inferior to the inhibitory effects of ribavirin (Figure 2D). Taken together, OMT displayed different ways to alleviate RSV infection in HEp-2 cells. RSV replication was restrained by OMT especially via inhibiting virus biosynthesis and virus penetration. Taken together, OMT displayed different ways to alleviate RSV infection in HEp-2 cells. RSV replication was restrained by OMT, especially via inhibiting virus biosynthesis and virus penetration.

### 2.2. Preparation and Characterization of CS-Coated OMT Liposomes

In our previous reports, we prepared a CMCS-coated OMT liposomes via a pH-gradient method that constituted soybean phospholipid (SPC), cholesterol (Chol), polyoxyethylene stearate (SPEG) and octadecyl amine (ODA) for pulmonary emphysema treatment [22]. However, a fast release of OMT was detected regardless of CMCS coating. The instability of SPC was proposed to contribute to the leakage of OMT, and the usage of saturated lipids might be a solution [17,27]. Therefore, the OMT liposomes prepared by SPC, DPPC/SPC and DPPC/HSPC were formulated and characterized (Figure S2). The particle size of liposome increased along with the percentage of saturated lipids-DPPC and HSPC. An obviously retarded drug release was observed in the OMT liposomes prepared by DPPC/HSPC. Hence, DPPC/HSPC/DPPG/Chol/DSPE-PEG at the weight ratio of 20:1:5:10:1 was used to prepare Lipo/OMT and used in the following studies.

CS coating was suggested to confer muco-adhesivity to nanoparticles and mediate cellular uptake [28,29,30,31]. Both high-molecular-weight CS (HCS) and low-molecular-weight CS (LCS) were coated on the liposomes at various weight ratios (Figures S3 and S4). As a matter of fact, HCS showed a strong combination with liposomes, leading to increased particle size. A large amount of precipitation and a liquid–solid interface were observed after standing still for 4 h until the HCS:liposome weight ratio reached 2.5:1. Intriguingly, the particle size of LCS-coated liposomes reached the highest particle size (337 nm) at a LCS:liposome ratio of 1:10 (*w*/*w*) and sharply decreased to 234 nm at 1:5 (*w*/*w*), and then a slightly variation was observed. Given that the high ratio of HCS resulted in a high viscosity of the solution that failed in effective inhalation, the LCS was used in the following study. The zeta potential was decreased until the LSC:liposome ratio reached 1:2.5 *(w/w)*, and a larger SD value was measured at a higher LSC:liposome ratio, which suggested that LCS reached saturated adsorption on liposomes. Therefore, LCS was coated on the surface of Lipo/OMT at the weight ratio of 1:2.5 to receive Lipo/OMT@CS.

**Figure 3:**
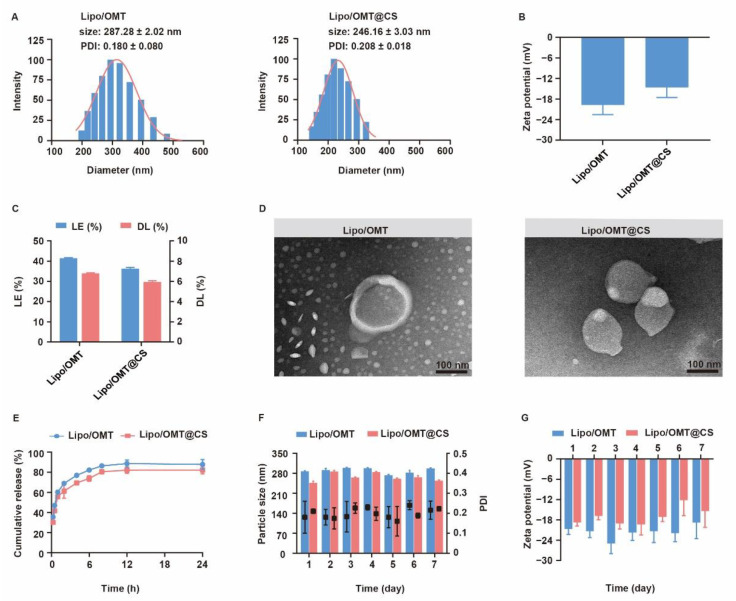
Characterization of OMT liposomal preparations. (**A**) Evolution of particle size, PDI and (**B**) zeta potential of OMT liposomal preparations. (**C**) Drug loading efficiency (LE) and drug loading capability (DL) of OMT liposomal preparations. (**D**) Representative TEM images of Lipo/OMT and Lipo/OMT@CS (scale bar 100 nm). (**E**) In vitro release behavior of OMT from liposomal preparations in pH 7.4 PBS. (**F**) The variation in particle size, PDI and (**G**) zeta potential of OMT liposomal preparations, at 4 °C, for 7 days. All the data were expressed as mean + SD (*n* = 3).

The physiochemical properties of OMT liposomal preparations were characterized and the stability was evaluated for one week (Figure 3). Lipo/OMT and Lipo/OMT@CS are 287.28 ± 2.02 nm and 246.16 ± 3.03 nm, respectively (Figure 3A). Small PDI value indicated good polydispersity of OMT liposomes. After CS coating, the zeta potential of liposomes increases from −19.63 ± 2.92 mV to −14.54 ± 3.04 mV, which remains negative (Figure 3B). The morphology of OMT liposomes were observed by transmission electron microscope (TEM). Lipo/OMT possesses spheric morphology with a clear layer outside that divides the particle into two parts, whereas irregular particles without an interface were observed in the Lipo/OMT@CS sample (Figure 3D), confirming the successful coating of CS on the liposomes. The loading efficiency (LE) and drug loading capacity (DL) of OMT liposomes were approximate 40% and 6%, respectively (Figure 3C). Drug release behavior was studied in the pH 7.4 medium (Figure 3E). More than 80% of OMT was released from liposomes at 12 h, and CS slightly retarded drug release from 2 h. The stability of OMT liposomes was evaluated in terms of particle size and zeta potential, at 4 °C, for 7 days (Figure 3F,G). No obvious fluctuation on the size and zeta potential was detected, which indicated the good stability of OMT liposomal preparations in vitro. Collectively, the CS coating on the periphery of liposomes decreased the surface charge without affecting the stability, while it exhibited a limited effect on retarding the release of OMT, which was likely due to the huge intermolecular channels of CS.

### 2.3. Stability and Penetration Behavior of CS-Coated Liposomes in Mucin Media

**Figure 4:**
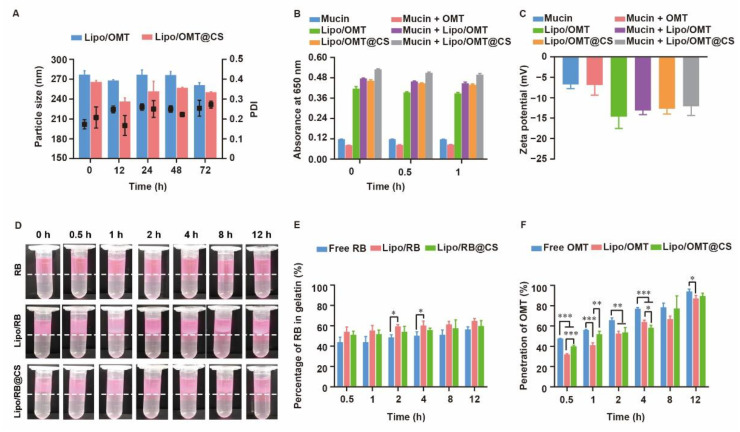
Stability and penetration capability of OMT/RB liposomal preparations in mucin media. (**A**) Particle size of OMT liposomal preparations in mucin. (**B**) Absorbance at 650 nm and (**C**) zeta potential of OMT liposomal preparations alone or in a mucin solution. (**D**) Visual observation of RB liposomes penetrations through the mucin layer at different time point (dotted line indicated the interface between mucin and gelatin layer). (**E**) The percentage of RB in gelatin layer. (**F**) Penetration percentage of OMT through mucin layer in transwells. All the data were expressed as mean + SD (*n* = 3). * *p* < 0.05, ** *p* < 0.01, *** *p* < 0.001.

The high stability of OMT-loaded liposomes in pulmonary-relevant fluids is the prerequisite of subsequent cellular uptake and drug release by the epithelial cells, since the aggregation or dissociation of liposomes would seriously restrict the pharmacological effects of OMT. As mucin play an important role in the mucus, the size variation of OMT-loaded liposomes in mucin media was monitored. As shown in Figure 4A, a slight fluctuation on diameter was observed in Lipo/OMT within 48 h, while a sharp decrease in the diameter of Lipo/OMT@CS was detected after 12 h incubation with mucin, thereafter re-increasing at 24 h. CS has been reported to show muco-adhesivity via the interaction between its amino groups and sialic acid and sulfated sugar in the mucin chains [28,32,33]. It was reasonable to suggest that the negatively charged Lipo/OMT prevented the interference from mucin, and the CS coating mediated the electrical compression and adhesion from mucin. To investigate the interaction between mucin and liposomes, the absorbance of liposomes in mucin at 650 nm was detected (Figure 4B). A similar absorbance of mucin and mucin + OMT was observed. Upon incubation, the absorbance of liposomes + mucin approximately equals to the sum of the absorbance of liposomes and mucin, indicating the poor tendency in the interaction between liposomes and mucin regardless of the CS coating. Correspondingly, no variation in the zeta potential of the liposomes was exhibited after incubation with mucin (Figure 4C).

For further assessment on the potential diffusion of liposomes through the airway mucus, two in vitro models were used to evaluate the penetration of rhodamine B (RB) liposomal preparations and OMT liposomal preparations. A two-layer model that consisted of a mucin upper layer and a gelatin sublayer was used to directly evaluate the penetration capability of the hydrophilic drug and its liposomes (Figure 4D,E). Both the visual observation and the quantitation of the percentage of RB in gelatin demonstrated a fast penetration at the initial 0.5 h, and no obvious differences were detected among all the groups. Meanwhile, a transwell model consisted of upper chamber filled with mucin and lower chamber filled with PBS was used to evaluate the penetration capability of OMT liposomal preparations (Figure 4F). Approximately 50% OMT was detected in the well at 0.5 h, and 100% penetration was achieved at 12 h. Intriguingly, free OMT showed even faster penetration than its liposomal preparations. It is reasonable to suggest that the hydrophilic drug might not be restricted by the mucus layer after inhalation, whereas the three-dimensional network formed by mucin will be an obstacle to nanosized particles. Although several reports have been pointing to the advantage of PEGylation on the deep and fast penetration of nanoparticles across mucus via reduction in hydrophobic and electrostatic interactions with mucin and other proteins, the high negative potential and strong steric hindrance of PEG shell might impede the cellular uptake after mucus penetration [34,35]. Hence, LCS was used in this study to increase the interactions between OMT liposomes and targeted cells without alteration on the surface electrical property, since positively charged liposomes might strongly induce interactions with mucin to form aggregation. In addition, the different penetration percentage between the two models might result from the resistance of high concentration of gelatin.

### 2.4. Biodistribution of RB Liposomal Preparations after Inhalation

**Figure 5:**
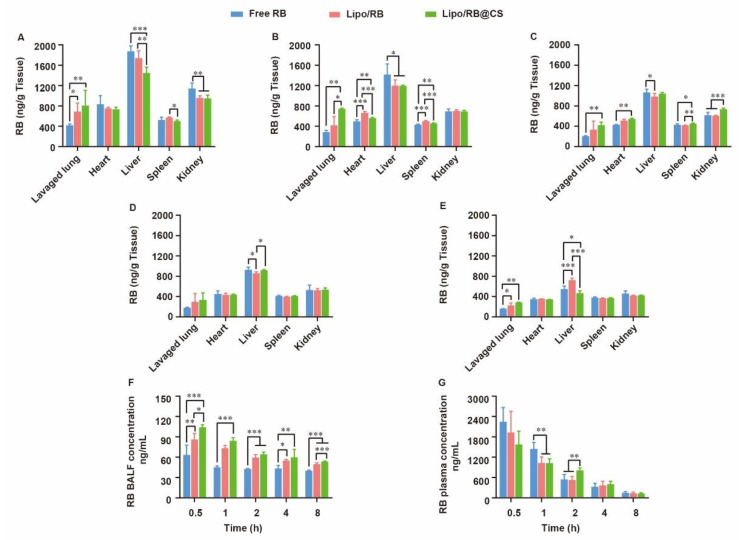
Biodistribution of RB in mice at (**A**) 0.5 h, (**B**) 1 h, (**C**) 2 h, (**D**) 4 h and (**E**) 8 h after inhalation of RB formulations and the concentration of RB in (**F**) BALF and (**G**) plasma. All the data were expressed as mean + SD (*n* = 6). * *p* < 0.05, ** *p* < 0.01, *** *p* < 0.001.

For lung infection treatment, pulmonary distribution and retention closely related to the therapeutic effects and systemic escape of drugs might lead to undesirable side effects. To discover the effects of liposomal carrier and CS coating on the biodistribution of hydrophilic drug, RB liposome (Lipo/RB) and CS-coated Lipo/RB (Lipo/RB@CS) were prepared, and the concentration of RB in lung, heart, liver, spleen, kidney, bronchoalveolar lavage fluid (BALF) and plasma were quantified at 0.5 h, 1 h, 2 h, 4 h and 8 h after inhalation (Figure 5). Half an hour after inhalation, the free RB group showed significantly higher concentration of RB in liver and kidney, and lower concentration in lung and BALF than that of the liposomal groups. Lipo/RB@CS showed the highest concentration of RB in both BALF and lung tissue within 1 h. No significant difference was detected between the Lipo/RB and Lipo/RB@CS after 2 h in lung. We can conclude that liposomes enhanced the accumulation of RB in lung, especially with CS coating.

Given that faster mucus penetration behavior of hydrophilic drug was observed in vitro (Figure 4), liposomal carrier and CS coating might contribute to the retention of RB in BALF. Furthermore, the higher accumulation of RB in lavaged lung of the Lipo/RB@CS group might result from the increased cellular uptake mediated by CS. The low BALF retention and high plasma concentration of RB in free RB group is closely related to fast diffusion of the hydrophilic drug though mucus layer, which might facilitate the penetration of RB through blood–gas barrier at the trachea and the alveolar sacs resulting in higher plasma concentration. As previously reported, the large surface area, highly permeable bio-membrane, and good blood supply of the alveolar region are favorable for rapid absorption of drugs [36]. However, it is not favorable for the local treatments on pulmonary infection. CS-coated liposomes have the potential to increase the lung accumulation and reduce the systemic and extrapulmonary exposure of the hydrophilic drug, which provides the foundation for the alleviative effects of OMT on RSV-induced lung infection.

### 2.5. Alleviative Effects and Mechanisms of OMT Liposomes on RSV-Infected Mice

**Figure 6:**
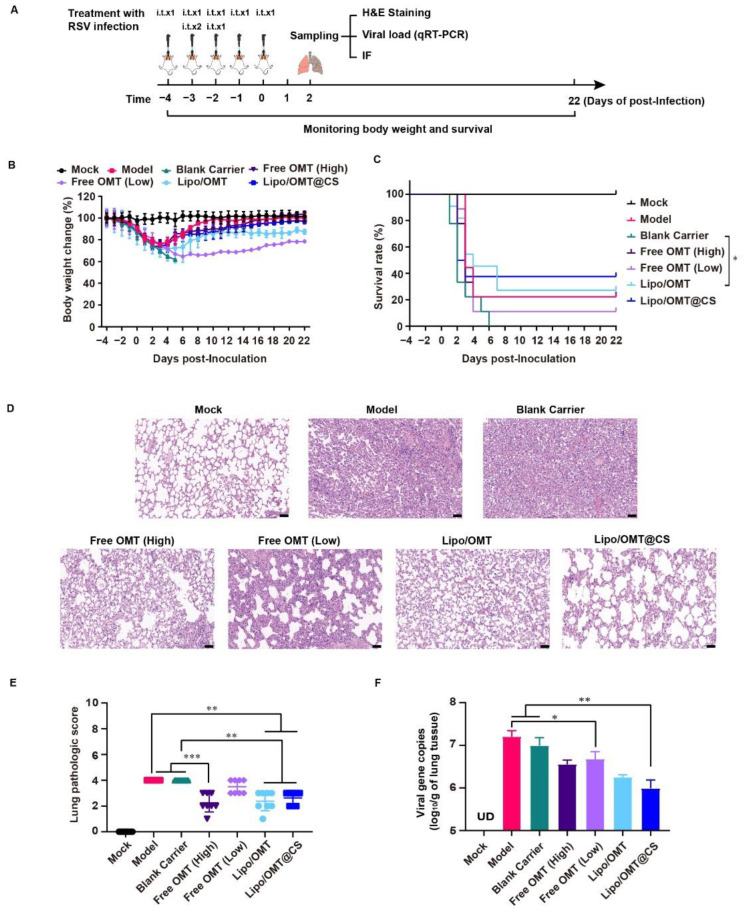
Alleviative effect of OMT liposomal preparations on RSV-infected mice. (**A**) Schematic illustration for the evaluation of alleviative effect of OMT liposomal preparations on RSV-infected mice. (**B**,**C**) Body weight change and survival rate of Mock (*n* = 8 at beginning), Model (*n* = 9 at beginning), Blank Carrier (*n* = 9 at beginning), Free OMT (High) (*n* = 9 at beginning), Free OMT (Low) (*n* = 9 at beginning), Lipo/OMT (*n* = 11 at beginning), and Lipo/OMT@CS (*n* = 8 at beginning). The timepoint of 22 DPI was set as end point of recording. Data for body weight change are expressed as mean ± SD. (**D**) H&E staining of pathologic sections of lung tissues for each group at 2 DPI (scale bar = 50 μm). (**E**) The lung pathologic scores basic on the H&E staining were double-blinded and accessed by anonymous pathologists and statistically processed. Data are expressed as mean ± SD (*n* = 8). (**F**) The qRT-PCR results for viral gene copies of lung BALF samples. Data are expressed as mean + SD (*n* = 3). UD: under detectable. * *p* < 0.05, ** *p* < 0.01, *** *p* < 0.001.

In order to evaluate the potential alleviative effects of OMT liposomal preparations in vivo, we introduced the lethal RSV infection mice model developed by our group [37]. As shown in Figure 6A, except for the Mock mice, the rest of the mice received a 300 μL GZ08-18 inoculation via inhalation within 2 days to establish the lethal RSV infection model. In the meantime, Blank Carrier mice were given CS-coated liposome. Free OMT mice were given 5 mg/kg or 20 mg/kg OMT solution as low dosage group and high dosage group, respectively. Lipo/OMT and Lipo/OMT@CS mice were inoculated with OMT liposomal preparations at an equivalent OMT dosage of 5 mg/kg. Mock and Model mice were given saline instead of drugs. The treatments were all conducted intra tracheal once per day for 5 consecutive days. The sampling of lungs for virus titration, hematoxylin and eosin (H&E) staining, and immunofluorescence assay was implemented at the 2nd day post-inoculation (DPI), and the rest of mice were kept till 21 DPI for daily weight and survival recording. The safety of OMT liposomes and blank carriers on the normal mice was implemented before the treatment. No mortality was observed after treatment by the OMT liposomes and blank carriers followed the same schedule in Figure 6A without RSV infection.

Combining the results of Figure 6B,C, Blank Carrier mice showed the worst inhibitory effect against the virus challenge, indicated by the loss of weight from −4 to 5 DPI, and the entire group died off at 6 DPI. However, two of nine Model mice survived the RSV lethal infection. The rest of the groups all had survivor (survivors) and weight curves that demonstrated the recovery after sharp declines which almost happened from 2 to 6 DPI. The survival rates of the Model, Blank Carrier, Free OMT (High), Free OMT (Low), Lipo/OMT, and Lipo/OMT@CS groups were 22.22% (2/9), 0 (0/9), 22.22% (2/9), 11.11 (1/9), 27.27% (3/11), and 37.5% (3/8), respectively. Compared with the Blank Carrier group, the Free OMT, Lipo/OMT, and Lipo/OMT@CS showed a protective effect against lethal RSV infection to mice, especially for Lipo/OMT@CS. However, no statistical difference was shown between Lipo/OMT@CS and other groups. Although a higher mortality and weight loss in the Blank Carrier group was observed than that in the Model group, fewer than three survivors in the Model, Blank carrier and Free OMT groups might be seen as a survivorship bias. Hence, a larger sample size is necessary in future studies.

Furthermore, the H&E staining confirmed that Model and Blank Carrier mice suffered from severe acute viral pneumonia indicated by alveolar structure destruction, leukocytes and cytokines infiltrations, congestion and edema of small vessels, etc., (Figure 6D). Although partial destruction of alveolar structure and enhanced thickness of alveolar wall were observed in free OMT, Lipo/OMT, and Lipo/OMT@CS groups, the basic alveolar structure was maintained, which was essential for O_2_/CO_2_ exchange, and fewer leukocytes and cytokines infiltrations were shown, resulting in a lower possibility of fatal cytokine storm. The H&E staining slides were further evaluated by pathologists (Figure 6E) according to the inflammation and tissue damage scoring system as previously reported [38]. In addition, the qRT-PCR results presented the significantly lower viral gene copies in BALF samples of Lipo/OMT@CS mice in comparison with Model and Blank Carrier. The Free OMT (Low) group showed significantly decreased viral gene copies compared with Model group as well. However, no significant difference was observed between Lipo/OMT@CS and other OMT preparations (Figure 6F).

**Figure 7:**
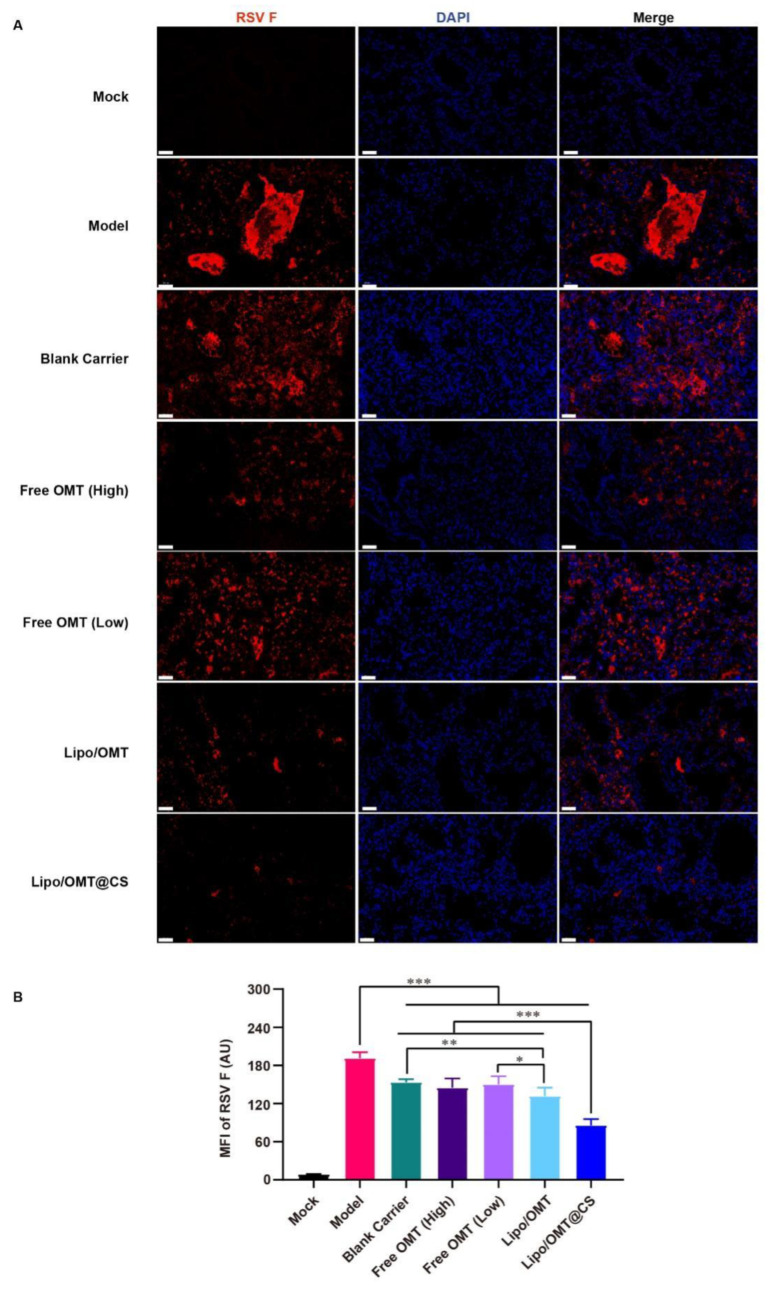
Immunofluorescence assay for OMT liposomal preparations on RSV-infected mice. (**A**) The representative images of each group were captured by confocal microscope (Scale bar = 50 μm). (**B**) Statistical result for the mean fluorescence intensity (MFI) of RSV F that calculated by Image J software from 8 equal sized images. All the data were expressed as mean + SD (*n* = 8). * *p* < 0.05, ** *p* < 0.01, *** *p* < 0.001.

Since F protein is one of viral membrane proteins which fuse the virus and cell membrane or infected cell membranes together, the expression of F protein demonstrates the attachment and entry capability of the virus. Hence, the immunofluorescence assay was conducted to show the expression of RSV F protein (Figure 7A). A huge amount of viral protein was found in the lumen of lung tissue in Model mice. The pulmonary lumen and interstitial were filled with RSV F protein in Blank Carrier mice as well. Similarly to Blank Carrier, scatter distribution of RSV F protein was also found in free OMT groups. However, less viral protein was shown in Lipo/OMT, especially in Lipo/OMT@CS mice. Compared with the results of gene copies (Figure 6F), a remarkably low level of viral protein was found in Lipo/OMT@CS, it being the lowest level among all the groups (*p* < 0.001, Figure 7B). In accord with the in vitro anti-RSV effects on HEp-2 cells (Figure 2), the main therapeutic mechanisms of OMT on RSV-infection might be attributed to its potential inhibitory effects on virus penetration and biosynthesis. Collectively, the alleviative effects of OMT on the lethal RSV infection of mice were endorsed, and Lipo/OMT@CS improved the preventive and therapeutic effects of OMT to some extent. The development and optimization of more effective inhalable liposomal carriers for pulmonary delivery of OMT is highly in need.

## 3. Materials and Methods

### 3.1. Materials and Reagents

Oxymatrine (OMT, purity ≥ 98%, lot no. C10572744), rhodamine B (RB, purity ≥ 99%, lot no. C10006604), and chitosan (CS, 800~1000 Da, lot no. C13207154) were offered by Macklin Biochemical Co., Ltd. (Shanghai, China). Cholesterol (Chol, purity ≥ 92.5%, lot no. WXBB1238) was purchased from VetecTM reagent grade (Shanghai, China). 1,2-dipalmitoyl-sn-glycero-3-phosphocholine (DPPC, lot no. CC0657) was provided by Corden pharma Switzerland LLC (Liestal, Switzerland). Hydrogenated soybean phosphatidylcholine (HSPC, lot no. B60455) and 1,2-dipalmitoyl-sn-glycero-3-phospho-(1′-rac-glycerol) (DPPG, lot no. B90497) were supported by A.V.T Pharmaceutical Co., Ltd. (Shanghai, China). 1,2-distearoyl-sn-glycero-3-phosphoethanolamine-N-methoxy (polyethylene glycol) (DSPE-PEG_2K_, lot no. RS0200412) was purchased from Xi’an ruixi Biological Technology Co., Ltd. (Xi’an, China).

### 3.2. Cells, Virus, and Animals

HEp-2 cells (CCL-23, ATCC, United States) were cultured under 5% fetal bovine serum (FBS) and 1% penicillin/streptomycin (P/S) supplemented with DMEM/F12 GlutaMax-I (10565, Gibco Ltd., Grand Island, NY, USA). GZ08-18, a highly virulent mouse-adapted RSV strain, was developed and stocked in our lab [37], and it was cultured under 2% FBS serum supplemented with DMEM/F12 GlutaMax-I. The cultured GZ08-18 was stock under −80 °C and viral titer was titrated by TCID_50_ assay and calculated by Spearman–Karber method.

Eight-month-old retired breeder BALB/c female mice (25–35 g) were supplied by the Experimental Animal Center of Guizhou Medical University. All animal care and experiments have been approved by the Animal Welfare and Ethics Committee of Guizhou Medical University (No: 1800020). Mice were raised at room temperature (23 ± 2 °C) and constant humidity (45 ± 10%) in an SPF condition. One week before starting experiments, animals were placed for adaptation.

### 3.3. Effects of OMT on Coculture of RSV and HEp-2 Cell

To evaluate the intervention of OMT on virus biosynthesis, GZ08-18 dilution with a multiplicity of infection (MOI) of 0.05 was added to HEp-2 cells for 1 h at 37 °C, then we discarded the virus dilution, washed the cells once with PBS, removed unadsorbed viruses, and added cell maintenance solutions containing different concentrations of OMT to culture for 72 h. To evaluate the inhibitory effects of OMT on virus penetration, GZ08-18 dilution with MOI of 0.05 was added to Hep-2 cells and cultured, at 4 °C, for 2 h, then we discarded the virus solution, washed the cells once with pre-cooled PBS, added different concentrations of OMT solutions, and cultured at 37 °C for 2 h. The OMT solution was discarded, and the cell maintenance solution was added to incubate cells for 72 h. To evaluate the inhibitory effects of OMT on virus attachment, pre-cooled OMT solutions and GZ08-18 diluent with MOI of 0.05 were added to Hep-2 cells, for 2 h, at 4 °C, then we discarded the OMT solution and virus diluent, washed the cells once with PBS, and removed unabsorbed OMT/virus. The cell maintenance solution was added to the cell and cultured for 72 h. To evaluate the preventive protective effects of OMT, different concentrations of OMT were added to the cells, incubated at 37 °C for 4 h, then we washed the cells once with PBS, added GZ08-18 dilution with MOI 0.05 for 1 h, at 37 °C, and then we washed the cells with PBS once and added the cell maintenance solution to incubate cells for 72 h. Meanwhile, ribavirin was used as the positive drug to coculture with virus and HEp-2 cells according to above experiments as the substitution for OMT.

### 3.4. Preparation of CS-Coated OMT Liposomal Preparations

OMT liposomal preparations were prepared using the pH-gradient method as reported previously [19]. In brief, DPPC, HSPC, DPPG, Chol, and DPEG-PEG2K were dissolved in a mixture of methanol and trichloromethane at a mass of 20:15:5:10:1 and transferred to a round bottom flask. Then, the solvent was removed by rotary evaporation (RE52CS, Shanghai Yarong biochemical instrument, Shanghai, China) to form thin films. The lipid film was resuspended with 0.15 mol/L citric acid solution (pH 1.9), and then incubated in water bath for 30 min, at 50 °C. Next, film was hydrated using a water bath ultrasonic apparatus for 2 min. The obtained liposomes were filtered through 0.45 μm filter. Additionally, the pH was then adjusted to 8.0 by sodium carbonate solution (0.3 mol/L), followed by coculture of OMT and liposomes for 10 min, at 50 °C, which were then cooled with cold water immediately. For the preparation of Lipo/OMT@CS, CS was coated on liposomal surface through electrostatic adsorption. Appropriate amount of CS (100 mg/mL) was dissolved under stirring in distilled water and added dropwise to the freshly prepared Lipo/OMT solution under stirring at 500 rpm. Ultimately, the free OMT was removed via dialysis against PBS to obtain Lipo/OMT and CS-coated OMT liposomal preparations.

The RB-encapsulated liposomes were prepared via a film dispersion method. Briefly, a thin lipid film was cocultured with 4 mL RB solution at 0.5 mg/mL and ultrasonicated. The Lipo/RB was obtained after passing through 0.45 μm filter. Lipo/RB@CS were obtained after modification of CS via the same methods described above.

### 3.5. Characterization of OMT Liposomal Preparations

The morphology of Lipo/OMT and Lipo/OMT@CS were observed by transmission electron microscope (TEM, Tecnai 12, Philips, Holland, Amsterdam, The Netherlands). The particle diameter, polydispersity (PDI), and zeta potential of Lipo/OMT and Lipo/OMT@CS were measured by NanoBrook 90Plus PALS (Brookhaven, GA, USA). The concentration of OMT in liposomal preparations was measured by high performance liquid chromatography (HPLC), which used Shimadzu LC-16 (Suzhou, China) with a UV detector (SPD-16, Shimadzu, Suzhou, China) using a reversed phase column (Hanbon, 4.6 × 250 mm, 5 μm, Huaian, China), at 30 °C. A mobile phase consisted of acetonitrile and 0.2% triethylamine (8: 92, *v/v*) at a flow rate of 1 mL/min and detecting at the wavelength of 210 nm. The loading efficiency (LE) and drug loading capacity (DL) were calculated according to Equations (1) and (2).

(1) $$\mathrm{LE}\;(\%)=\frac{W_{\mathrm{drug}\;\mathrm{loaded}}}{W_{\mathrm{total}\;\mathrm{Liposome}}}\times 100$$

(2) $$\mathrm{DL}\;(\%)=\frac{W_{\mathrm{drug}\;\mathrm{loaded}}}{W_{\mathrm{drug}\;\mathrm{added}}}\times 100$$

where W_drug loaded_ is the drug encapsulated into liposomes, W_total liposome_ is the mass of liposome with drug encapsulated, and W_drug added_ is the drug added in the preparation of liposomes.

In vitro release profile was measured using the dialysis method. Dialysis bag (MWCO, 14 KDa) holding 1 mL OMT liposomal preparations were immersed in 15 mL of PBS in a capped bottle and incubated, at 37 °C, with stirring at a speed of 50 rpm. At predetermined time point, 500 μL medium was withdrawn and replenished with fresh PBS at the same volume. The amount of OMT released into the medium was determined using the HPLC method described above. To determine the stability of formulations, OMT liposomal preparations were kept in an incubator, at 4 °C, for one week. The particle size, PDI and zeta potential were measured over the storage periods.

### 3.6. In Vitro Assessment of the Interactions between Liposomal Preparations and Mucus

#### 3.6.1. Stability of OMT Liposomal Preparations in Mucus

Stability of OMT liposomal preparations in mucin solution was evaluated. Mucin powder was dispersed in PBS and stirred overnight, then the dispersion was centrifuged at 3500× *g* for 10 min to receive a 0.08% (*w/v*) mucin supernatant solution. For the analyses, 200 μL of OMT liposomal preparations were added to 2 mL of medium and incubated, at 37 °C, for 72 h. At selected time intervals, the particle size of OMT liposomal preparations were monitored. The variation on particle size were considered as indicative of disassembly/aggregation state.

#### 3.6.2. Mucoadhesive Tendency

The mucoadhesive tendency of OMT liposomal preparations were assessed by turbidimetric measurement as previously reported [39,40]. Briefly, mucin solutions (0.08%, *w/v*) and OMT liposomal preparations were mixed at volume ratio of 1:1 and vortexed for 1 min. The absorbance at 650 nm of the mixtures was measured at 0, 30 and 60 min after incubation, at 37 °C, by the ultraviolet spectrophotometer (UV-2700, Shimadzu, Japan). The mucin solution and the liposomal preparations dispersions in PBS were analyzed as controls. For the measurement of zeta potential, the mucin solutions (0.08%, *w*/*v*) and mixture samples were diluted in PBS (1:4, *v*/*v*) and measured.

#### 3.6.3. Penetration of RB Liposomal Preparations through Mucus

Mucus penetration of RB-encapsulated liposomal preparations were evaluated by the in vitro mucus model [40,41]. One milliliter of 10% (*w*/*v*) hot gelatin solution was prepared, placed into a centrifuge tube, hardened at room temperature, and placed in a refrigerator, at 4 °C, until use. For the mucus penetration study, 0.5 mL of mucin solution (0.08%, *w/v*) was added above the gelatin solution and 125 μL of free RB solution and RB liposomal preparations were added on the mucus layer and maintained, at 37 °C. After 0.5 h, 1 h, 2 h, 4 h, 8 h, and 12 h incubation, the mucus layer was transferred to 1 mL volumetric flask. RB in the mucus layer was extracted with methanol and measured by a fluorescence spectrophotometer (Cary Eclipse, Varian, USA) at λ_Ex_/λ_Em_ = 550/580 nm. The percentage of RB penetrated through mucus was calculated according to Equation (3).

(3) $$Penetration\;percentage\;\left(\%\right)=\frac{Drug_{\mathrm{added}}-Drug_{\mathrm{mucus}}}{Drug_{\mathrm{added}}}\times 100\%$$

#### 3.6.4. Transwell Penetration of OMT Liposomal Preparations through Mucus

The transwell multiwall plates were applied to evaluate the penetration capacity of OMT liposomal preparations across mucus layer [42]. Briefly, 150 μL of mucin solution (0.08%, *w/v*) was transferred to the insert of transwell (polycarbonate membrane, 3 μm, 0.33 cm^2^). Then, 100 μL of free OMT and OMT liposomal preparations were gently added above the mucus layer. The insert of transwell was immersed in the acceptor chambers that were filled with 1 mL of PBS and incubated, at 37 °C. At the scheduled time intervals, 100 μL of samples were collected from acceptor chambers, and an equal volume of fresh PBS were replenished. The amount of OMT in the acceptor chambers was determined using the HPLC method, and the percentage of OMT penetrated through mucus was calculated according to Equation (3).

### 3.7. In Vivo Biodistribution of RB Liposomal Preparations

The distribution of RB liposomal preparations in organs were investigated according to the method reported previously [22]. Tracheal intubation (22G surflo^®^. I. V. Catheter, Terumo Corp, Biñan, Philippines) was used for the inhalation administration of free RB and RB liposomal preparations. Briefly, at specific time points, mice were anesthetized by isoflurane via inhalation anesthesia. Free RB solution and RB liposomal preparations were inhalation administered at an equivalent RB dose of 0.75 mg/kg, respectively. At 0.5 h, 1 h, 2 h, 4 h and 8 h after treatment and under anesthesia, blood samples were taken from the aorta abdominalis, and the plasma was obtained by centrifugation (3000 rpm, 10 min) and stored, at 4 °C, until analysis. After the euthanasia treatment, BALF and organs (heart, liver, spleen, lung, kidney) were obtained. The tissues were homogenized in PBS. Afterwards, ethanol was added to the plasma, BALF and organ homogenates at certain ratio to extract RB. Thereafter, the samples were centrifugated at 3000× *g* for 10 min, and the supernatants were used to measure the concentration of RB via a fluorescence spectrophotometer.

### 3.8. Treatments on RSV-Infected Mice

Based on lethal RSV-infected mouse models established by our laboratory as one regimen, 100 μL GZ08-18 culture medium (containing 1×10^10^TCID_50_ virus) was inoculated into mice via trachea, and three regimen (with 10–12 h interval) should be inoculated into pulmonary system of mice to establish the lethal RSV-infected mouse model. A total of 63 BALB/c mice were involved in the body weight change and survival experiments. They were randomly divided into 7 groups (n = 8–11 for each group): Mock, Model (virus control), Blank Carrier (blank preparation plus virus), Free OMT (High) (free OMT 20 mg/kg plus virus), Free OMT (Low) (free OMT 5 mg/kg plus virus), Lipo/OMT (5 mg/kg Lipo/OMT plus virus), and Lipo/OMT@CS (5 mg/kg Lipo/OMT@CS plus virus). The Mock mice were administered with saline as negative control. The Model mice were administered with saline plus virus. The Blank Carrier mice were administered with Lipo@CS solution.

The schematic experiment arrangement for exploration of the alleviated effect of OMT liposomal preparations was illustrated in Figure 6A. Briefly, the Mock, Model, Blank Carrier, Free OMT (High), Free OMT (Low), Lipo/OMT, and Lipo/OMT@CS were received saline, free OMT, or OMT liposomal preparations treatments for 6 consecutive days. All the groups except the Mock were infected with 300 μL GZ08-18 for 2 consecutive days. We define the day after last inoculation as the 1st day post-inoculation (1 DPI). The body weight and survival of each mouse were documented daily till 22 DPI.

### 3.9. Analysis of Bronchoalveolar Lavage Fluid (BALF)

At 2 DPI, the mice were properly anaesthetized and euthanized. Subsequently, the right lobe of lung was fully perfused with 800 μL DMEM/F12 GlutaMax-I (containing Vancomycin 20 μg/mL, Ciprofloxacin 20 μg/mL, Amikacin 0.05 μg/mL, Nystatin 50 μg/mL, and P/S 1%), and the BALF was obtained. After centrifugating the BALF sample under 4 °C, at 12,000 rpm for 10 min, the cell-free supernatant is transferred to sterilized enzyme-free 1.5 mL centrifuge tubes for subsequent qRT-PCR assay for viral load analysis. Briefly, the viral RNA was firstly extracted by using the TaKaRa MiniBEST Viral RNA/DNA Extraction Kit (9766, TaKaRa Ltd., Beijing, China) from BALF-derived cell-free supernatant. cDNA was generated by PrimeScript™ 1st Strand cDNA Synthesis Kit (6110A, TaKaRa Ltd., Bejing, China). The qRT-PCR was then performed with the TB Green^®^ Fast qPCR Mix kit (RR430A, TaKaRa Ltd., Beijing, China). We performed the RSV qRT-PCR absolute quantitative detection method established by our laboratory to quantitatively measure the viral copies via a standard curve of RSV *N* gene. Since *N* is the most conserved RSV gene across all known genetic clades, and is also expressed most abundantly during viral replication [43].

### 3.10. Histopathology Evaluation of Lung Tissue

At 2 DPI, the mice were properly anaesthetized and euthanized. The left lung lobe was collected and fixed immediately in 4% formaldehyde under 0.01 M phosphate buffer (pH 7.4). After dehydration and embedding in paraffin, the lung tissue was cut into 5.0 μm-thick sections and stained with hematoxylin and eosin (H&E) for evaluation of histopathology and of the severity of pneumonia. The slides were scored with a semiquantitative system according to the relative degree of inflammation and tissue damage (Table S1).

### 3.11. Immunofluorescence Experiment

The left lung lobe harvested at 2 DPI was processed into paraffin sections, and then the sections were then deparaffinized and rehydrated. After treatment, immersed the slides in EDTA antigen retrieval buffer for antigen retrieval. Because F protein is one of viral membrane proteins and the function of F protein is fusing RSV and cell membrane or infected cell membranes together. So its expression would demonstrate the attachment and entry capability of virus. Therefore, slides were blocked by 1% BSA and incubated with primary anti-RSV F antibody (sc-101362, Santa Cruz Biotechnology Ltd., Dallas, TX, USA). Then, slides were incubated with fluorescent secondary antibody. 4’,6-diamidino-2-phenylindole (DAPI) counterstained nucleus for 15 min, and then images were detected and collected by fluorescent microscope (ECLIPSE C1, Nikon, Tokyo, Japan) and confocal microscope (DS-U3, Nikon, Tokyo, Japan). Nucleus is blue by labeling with DAPI, and positive signals are red according to the fluorescent labels used.

### 3.12. Statistical Analysis

Data are presented as mean ± or + SD. The one-way ANOVA followed by post hoc Tukey test was used in the statistical analysis among multiple groups using GraphPad Prism 6. Comparisons between Mock and Model groups were assessed by unpaired, two-tailed Student’s t test. Variance test (F test) was implemented before the one-way ANOVA analysis and t-test to check whether the data met the normal distribution. The Kaplan–Meier method was used for survival analysis, and the Log-rank test was performed to analyze the differences among the survival curves. The Mann–Whitney test was applied for the pathologic scores analysis. Significance was defined as *p* values of less than 0.05.

## 4. Conclusions

In this study, we developed a CS-coated liposome to facilitate the alleviative effect of OMT on lethal RSV infection via improving the lung accumulation and retention of OMT to exert its anti-virus effects by inhibiting the penetration and biosynthesis of virus. We revealed that effective penetration through the blood–gas barrier leads to fast systemic distribution and short exposure at lung, instead of pulmonary mucus being the main barrier for OMT in the treatment of RSV infection. OMT liposomal preparations, especially Lipo/OMT@CS, reduced the virus replication and increased the survival rate of RSV-infected mice. Further investigation is warranted to elaborate the exact mechanisms of OMT on the alleviation of lethal RSV infection and to optimize the liposomal carriers.

## Data Availability

Not applicable.

## Supplementary Materials

The following supporting information can be downloaded at: https://www.mdpi.com/article/10.3390/ijms232415909/s1.
