# Supplementary material for: Development of Inhalable Chitosan-Coated Oxymatrine Liposomes to Alleviate RSV-Infected Mice
Source: Int J Mol Sci. 2022 Dec 14;23(24):15909. doi: 10.3390/ijms232415909 (PMC9786244; doi:10.3390/ijms232415909)
Supplement: Supplementary file 1 [file ijms-23-15909-s001.zip › ijms-2031680-supplementary.pdf]

Supporting Information

Development of Inhalable Chitosan-Coated Oxymatrine Liposomes to Alleviate RSV-Infected mice

Jianqing Peng[1,2], Qin Wang[1,2], Mingyang Guo[3], Chunyuan Liu[3], Xuesheng Chen[3], Ling Tao[1,2], Ke Zhang[1,3*], Xiangchun Shen[1,2*]

1 The High Efficacy Application of Natural Medicinal Resources Engineering Center of Guizhou Province, School of Pharmaceutical Sciences, Guizhou Medical University, University Town, Guian New District, Guizhou 550025, China

2 The Key Laboratory of Optimal Utilization of Natural Medicine Resources, School of Pharmaceutical Sciences, Guizhou Medical University, University Town, Guian New District, Guizhou 550025, China

3 The Key and Characteristic Laboratory of Modern Pathogenicity Biology, School of Basic Medical Sciences, Guizhou Medical University, University Town, Guian New District, Guizhou 550025, China

Figures

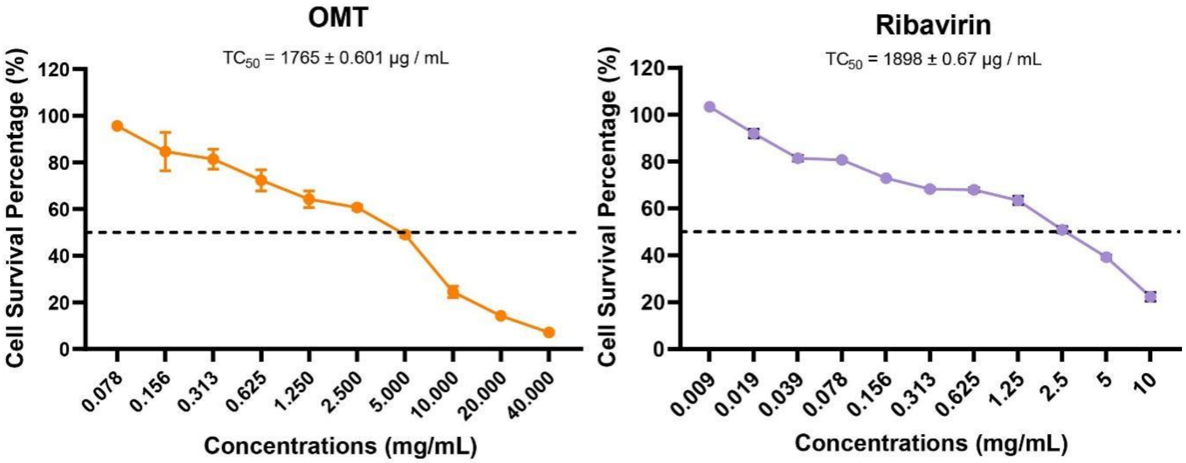

Figure S1. The cytotoxicity of OMT and ribavirin on HEp-2 cells.

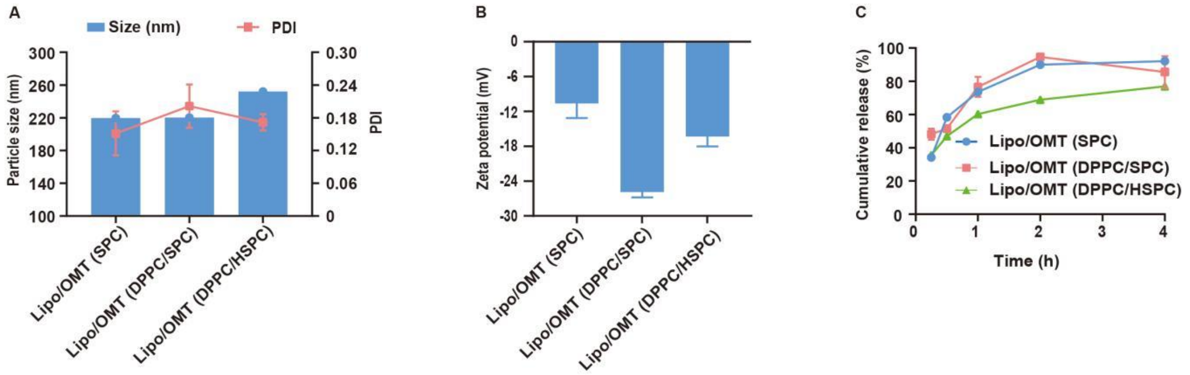

Figure S2. Optimization on the prescription of Lipo/OMT. (A) Particle size, PDI and (B) zeta potential of OMT liposomal preparations. (C) Release behavior of OMT from different liposomal preparations in pH 7.4 PBS. Each bar and dot represent the means ± SD (*n* = 3).

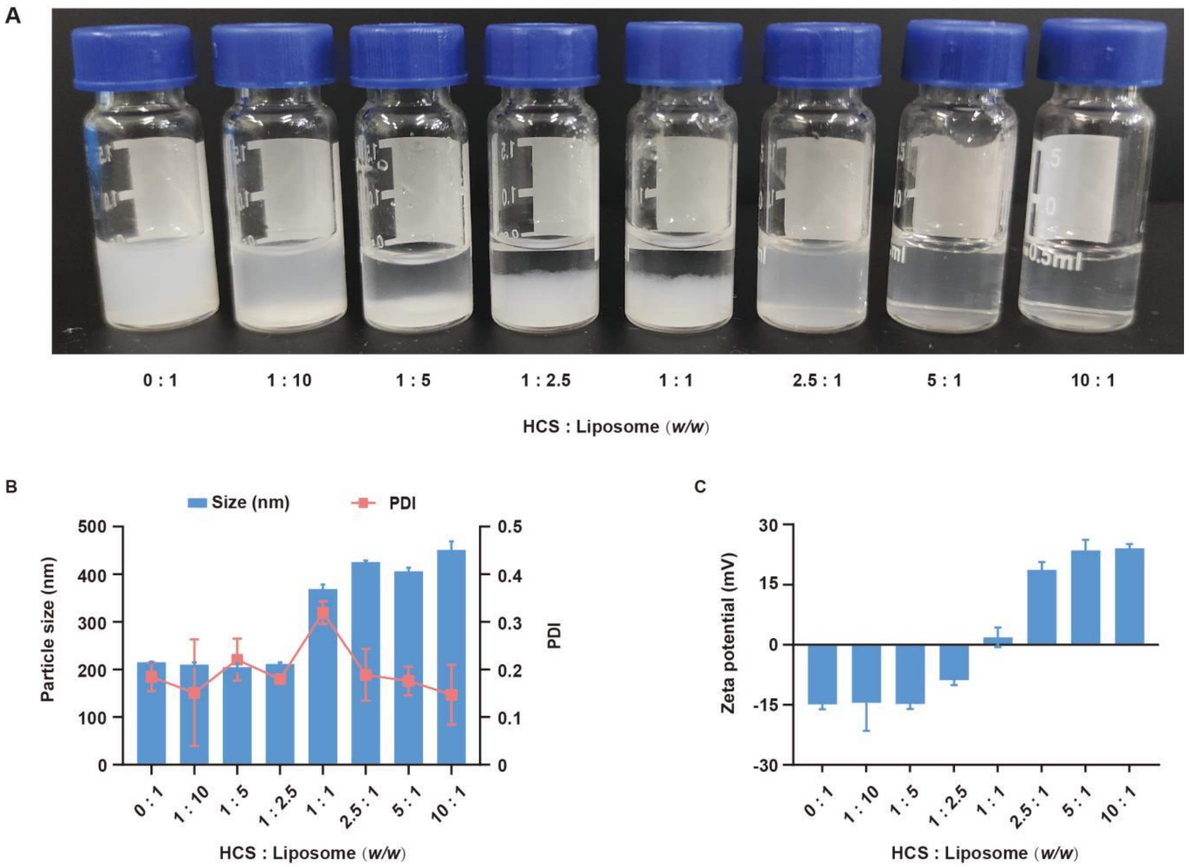

Figure S3. Characterization of HCS-coated OMT liposomes. (A) Appearance, (B) particle size/PDI, and (C) zeta potential of OMT liposomal preparations at various HCS : liposome weight ratio. Each bar represents the means ± SD (n = 3).

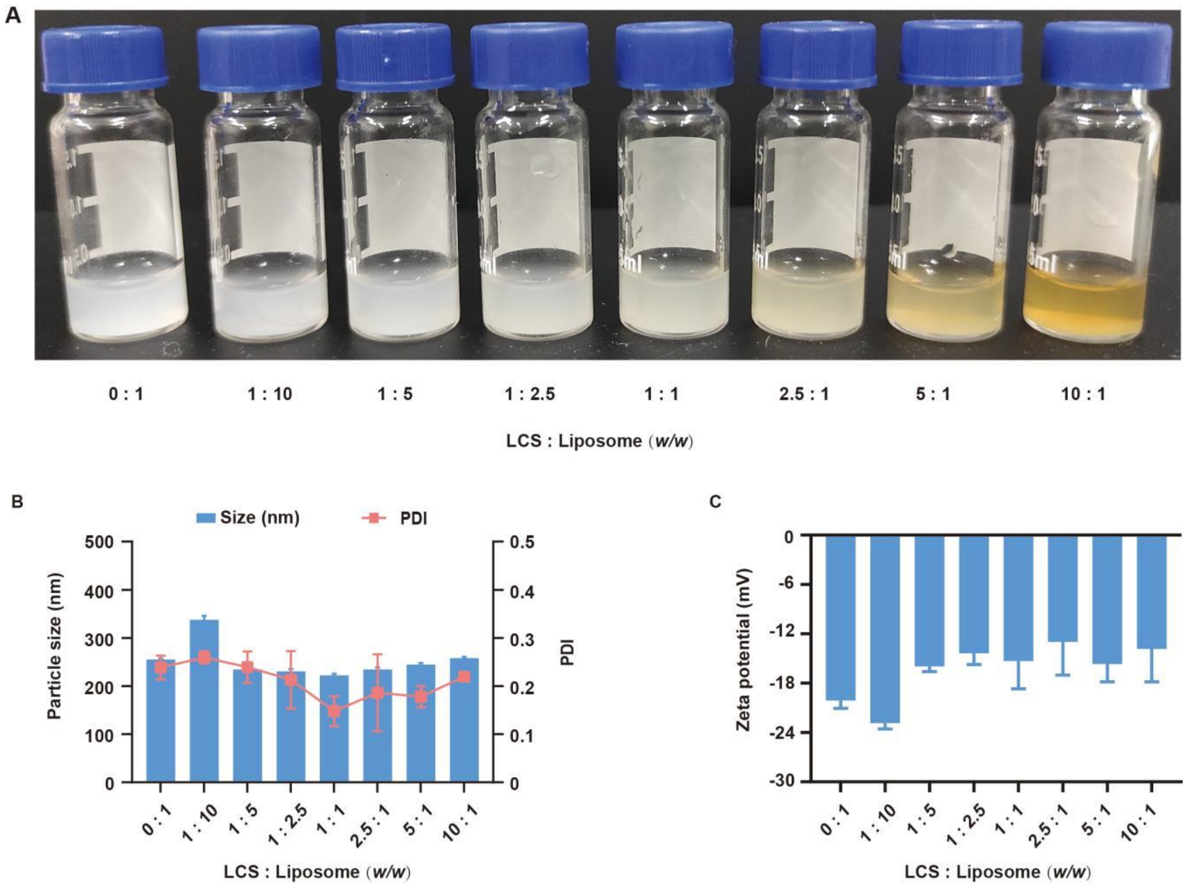

Figure S4. Characterization of LCS-coated OMT liposomes. (A) Appearance, (B) particle size/PDI, and (C) zeta potential of OMT liposomal preparations at various LCS : liposome weight ratio. Each bar represents the means ± SD (n = 3).

Table S1. Inflammation and tissue damage scoring system.

Scored	Relative degree of inflammation and tissue damage
0	No inflammation
1	Perivascular cuff of inflammatory cells
2	Mild inflammation (extending throughout 25% of the lung)
3	Moderate inflammation (25-50% of the lung)
4	Severe inflammation involving over one half of the lung
